# Antecedents of Compliance Intention and Its Impact on Waste Separation Behavior: Based on Rational Choice Theory and Deterrence Theory

**DOI:** 10.3390/bs13050424

**Published:** 2023-05-17

**Authors:** Sohee Kim

**Affiliations:** Division of Social Welfare and Child Studies, Daejin University, Pocheon-si 11159, Republic of Korea; niki88@daejin.ac.kr

**Keywords:** waste separation policy, waste separation behavior, rational choice theory, deterrence theory, compliance intention

## Abstract

With the rapid growth of the urban population, the development of production and consumption, and improved living standards, waste generation has increased over time. The first positive step to solve the problem of household waste is waste separation behavior. Studying the determinants that prompt individuals to comply with waste separation policy (WSP) is worthwhile. The author aims to offer an integrated view of individuals’ compliance with waste separation policy based on rational choice and deterrence theories. Survey data collected from 306 households in South Korea are used to test the research model using partial least squares analysis. The study shows that WSP compliance intention is motivated by the perceived benefit and perceived effectiveness of WSP. Furthermore, the results show that perceived deterrent severity and perceived deterrent certainty positively influence WSP compliance intention. The implications for theory and policymakers are discussed to facilitate waste separation behavior.

## 1. Introduction

Vast volumes of waste have been generated from households [1]. The rapid growth of household waste has become a critical global issue because it threatens human health and the environment if not correctly dealt with. Waste separation behavior is the first positive step to solving household waste and achieving a sustainable environment [2]. Separating waste at the source has become an essential component of waste management strategies for reducing waste in many countries [3]. Given the urgency of waste disposal problems, many governments have implemented waste separation policies to reduce disposal in landfills [4]. According to the report of the World Bank [5], the recycling rate of municipal solid waste in South Korea was 58%, Singapore 61%, Germany 47.8%, and the USA 34.6%.

Over the past decade, South Korea has been one of the fastest-growing OECD economies. The rapid economic growth of this country has been accompanied by significant pollution and waste disposal [5]. Due to the early establishment of the Waste Management Law in the 1980s, the total municipal solid waste generation declined by 36% in the 1990–2016 period (514.5 kg in 1985 to 384.9 kg in 2016) [6]. Seoul, a metropolitan in South Korea, is trying to promote sustainable residential waste separation behaviors. Although Seoul restricted waste disposal by mandating each type of waste to be disposed of, some residents’ unauthorized garbage dumping led to reeking and dirtier streets. Despite governments’ commitments to tackling the waste problem, the waste separation policy has not been successful due to a lack of individual voluntary participation [4,7].

Understanding what encourages people to comply with the waste separation policy is crucial to stimulate individuals’ participation in waste separation behavior. Most empirical research on waste separation behavior has been based mainly on the theory of planned behavior (TPB) and the attitude–behavior–context (ABC) theory. Previous research has focused on studying the costs of waste separation behavior at the individual level and has yet to consider the enforcement of public policy. However, many people are also ready to care more about waste separation behaviors than their immediate personal gains [8]. Citizens’ choices are informed by regulations other than self-interest or personal gains [9,10].

While the literature on waste separation behavior abounds, nearly all concerns the intention to comply with the waste separation policy [4,11]. In improving waste separation policy, understanding of the fundamental effects of mandatory policy on waste separation behavior is commonly underestimated. Studying the determinants that prompt individuals to comply with waste separation policy is worthwhile. Thus, this paper postulated that the psychological response to a mandatory waste separation policy could promote individuals’ intention to comply with the waste separation policy and lead to waste separation behavior.

To our knowledge, few studies have integrated rational choice theory and deterrence theory to understand household waste separation behavior. Further, even fewer investigations have been conducted to comprehensively examine the factors affecting compliance with household waste separation policy through a single framework. Hence, this study aims to offer an integrated view of individuals’ compliance with waste separation policy based on both rational choice theory [12,13] and deterrence theory [14,15].

The background of the research and hypotheses are presented in Section 2. The research methodology is described in Section 3, while the detailed results and discussion are described in Section 4. The conclusions are presented in Section 5.

## 2. Literature Review and Hypotheses

### 2.1. Research on Waste Separation Behavior

From the actor’s viewpoint, pro-environmental behavior is defined as behavior that is undertaken with the intention to benefit the environment [16]. This perspective highlights individuals’ intent as the cause of pro-environmental behavior, suggesting that individuals intend to comply with environmental policy. Waste separation behavior is an environmentally significant behavior in the private sphere and one of several distinct types of pro-environmental behavior, such as non-activist behaviors in the public sphere [17] and green practices in an organization [18].

Waste separation is defined as a process by which waste is separated into different elements operated manually in the household [2,19]. The primary purpose of waste separation is to lessen environmental damage and achieve environmental sustainability [20]. While most waste mitigation efforts are still required at the government and industry level, waste separation behavior at the household level is essential to reduce, recycle, and reuse waste [21]. These benefits include reduced waste disposal and collection costs, reduced waste picker problems, resource conservation, prolonging the lifespans of landfills, and less carbon emission [22].

Understanding the factors affecting individuals’ household waste separation behavior has been a research topic for many scholars in environmental literature. The literature offers various indicators that influence household waste separation behavior. These factors include the following: sociodemographic factors such as housing characteristics [20], psychological factors, economic factors [23], and political factors [24,25]. Specifically, most studies on psychological factors focus on perceived convenience and effort, state of knowledge and information, social norms, moral norms, attitude and environmental concern, habit, and system trust and community [3]. Furthermore, previous research on waste separation behavior has largely been based on a different theoretical framework, such as the theory of planned behavior (TPB) [26,27], norm activation model (NAM) [4,28], attitude–behavior–context model (ABC) [26,29], and dual-factor theory (DFT) [21].

### 2.2. Waste Separation Policy

With the rapid growth of the urban population, the growth of production and consumption, and improved living standards, waste generation has increased over time [30]. Four options to tackle the issues of the rapid expansion of waste generation include the following: landfill, incineration, recovery, and waste recycling [31]. Among these options, waste recycling has been regarded as the preferable option to tackle environmental issues [1] because it can reduce the waste of resources and mitigate the adverse effects of waste on the environment [32]. In fact, waste separation is a prerequisite of waste recycling [33] and is still a popular strategy for waste management in many countries [20].

For example, in South Korea, municipal waste disposal is divided into landfill, recycling, composting, and incineration [34]. Most municipal wastes were reclaimed in local or metropolitan landfills, and very little was recycled in the 1980s and before. However, due to the difficulty of obtaining more land for landfill sites, finding ways to reduce waste generation and increase waste recycling was crucial. Since 1995, in an attempt to reduce the quantity of waste and increase the rate of recycling, the South Korean government has implemented solid waste management (SWM) legislation and initiatives, including a volume-based waste disposal fees (VBWF) system, a volume-based food waste disposal fees (VBFWF) system, a deposit refund system, extended producer responsibility (EPR), and bans on problematic plastic items and packaging, leading to waste reduction since the early 1990s [6]. Since 1996, the amount of waste from Seoul going to landfill has fallen by almost 90%. In recent years, nearly 70% of Seoul’s household general waste has been recycled, with less than 10% going to landfill and the rest being processed at Seoul’s resource recovery facilities [35].

Recent years have witnessed the appearance of mandatory environment policy as a crucial factor affecting individuals’ waste separation behavior [4,36,37,38,39,40]. Hence, several governments encourage people to engage in waste separation behavior [41]. However, with the rising government regulation around the globe, household waste separation is no longer merely a voluntary behavior driven by personal attitudes. Despite municipal governments’ commitment to tackling the waste problem, only 15% of waste is recycled around the globe [42]. To increase waste recovery from urban waste, better household waste separation is needed, and active sustainable waste management has to be taken at the individual level [2,21,27,28].

However, while the literature on household waste separation behavior abounds, few studies concern the factors affecting individuals’ compliance with the waste separation policy. Given the growth of mandatory waste separation policy, studying the determinants of compliance with waste separation policy is worthwhile.

### 2.3. Compliance with Waste Separation Policy

Given the similarity between municipal environmental policy violations and criminal behavior in social settings, the theoretical perspectives developed in criminology literature could be adopted as the foundations for waste separation behavior research, including, but not limited to, rational choice theory [12,13] and deterrence theory [14,15].

Two relevant theories, i.e., rational choice theory and deterrence theory, will be integrated to increase our knowledge of waste separation policy compliance. To the best knowledge, prior research has yet to use both theories in a single study on waste separation behavior.

#### 2.3.1. Perceived Benefit and Perceived Effectiveness of Waste Separation Behavior

Rational choice theory, one of the criminological theories, is essentially a subjective expected utility theory, suggesting that an individual’s decision calculus to offend is based on the perceived or personal expectations of cost and benefit [43]. Thus, individuals are sensitive to the consequences of their behaviors and make reasoned decisions based on the cost–benefit analysis of the intended behaviors [12].

In the context of waste separation, individuals only participate in waste separation if the expected utility of waste separation exceeds that of conventional waste disposal [23]. This model identifies two considerations in an individual’s decision to adopt waste separation behavior in response to cost–benefit analysis, i.e., perceived benefit of waste separation behavior and perceived effectiveness of waste separation behavior. Perceived benefit (PB) refers to individuals’ personal belief in the positive impact of waste separation behavior on the environment [44]. Previous studies suggest that an individual aware of the benefit of waste separation behavior will engage in it [10,45]. Perceived effectiveness (PE) refers to individuals’ perceptions about the effectiveness of their ecological efforts in reducing environmental harms through waste separation behavior [46]. If an individual perceives more effective external motivation, the intention to perform a certain behavior will become stronger. Previous studies show that if an individual has a higher level of awareness of the positive consequences of waste separation behavior, the intention to perform such behavior would be higher [7,47]. Thus, higher perceived benefit and perceived effectiveness will likely lead to greater intention to comply with the waste separation policy. This study proposes the following research hypotheses:

**Hypothesis** **(H1).** *Perceived benefit of waste separation behavior positively influences intention to comply with the waste separation policy.*

**Hypothesis** **(H2).** *Perceived effectiveness of waste separation behavior positively influences intention to comply with the waste separation policy.*

#### 2.3.2. Perceived Deterrent Severity and Perceived Deterrent Certainty

Deterrence theory argues that criminal behavior results from a rational calculation of costs and benefits [12,48]. When individuals perceive that the costs of deviant behaviors outweigh the benefits, they will choose to comply with the law rather than engage in crime. The theory proposes that deterrence against certain deviant behaviors can prevent individuals from engaging in violations, suggesting the effect of formal sanctions in motivating individuals to follow public policies [4].

The theory assumes that individuals calculate the disutility of sanctions and try to minimize it by considering perceived deterrent severity and certainty [11,49]. Perceived deterrent severity (DS) refers to the deterrence’s harshness or the price to be paid for the crime [4,38]. Perceived deterrent certainty (DC) refers to the possibility that the deviant behavior will be discovered and a penalty imposed [4,38]. Individuals are expected to comply with public policies as the level of deterrent severity and certainty increases [49].

In the context of waste separation, deviant behaviors include inadequate separation, wrong location or container placement, and midnight dumping [4]. The corresponding deterrence includes fines, detention, and imprisonment. In this case, the benefits of not complying with waste separation policy are only time-saving and reducing daily chores. In contrast, the cost of violating the waste separation policy is much higher than the benefit. For example, illegal waste disposal in Seoul could result in a fine of up to KRW 300,000 (or approximately USD 250). Moreover, a surveillance camera on a power pole monitors illegal waste dumping. This deterrent effect will increase compliance with the waste separation policy. Thus, higher perceived deterrent severity and perceived deterrent certainty will likely lead to greater intention to comply with the waste separation policy. This study proposes the following research hypotheses:

**Hypothesis** **(H3).** *Perceived deterrent severity positively influences intention to comply with the waste separation policy.*

**Hypothesis** **(H4).** *Perceived deterrent certainty positively influences intention to comply with the waste separation policy.*

#### 2.3.3. Antecedents of Waste Separation Behavior

The theory of planned behavior assumes that individuals’ behaviors are influenced by intentions [50]. In the context of waste separation, previous studies provided empirical evidence that the intention of waste separation behavior forces waste separation behavior [19,51]. Thus, this study proposes the following research hypotheses:

**Hypothesis** **(H5).** *Intention to comply with waste separation policy positively influences waste separation behavior (i.e., compliance behavior).*

This study also included moral belief as a control variable positively affecting waste separation behavior. Moral belief is based on the perspective that what individuals regard as morally right or wrong affects their behavior [48]. In our context, moral belief refers to the degree to which individuals perceive a violation of the waste separation policy as morally unacceptable [27,52]. Previous studies indicate that moral belief is crucial to waste separation behavior [53,54].

**Figure 1:**
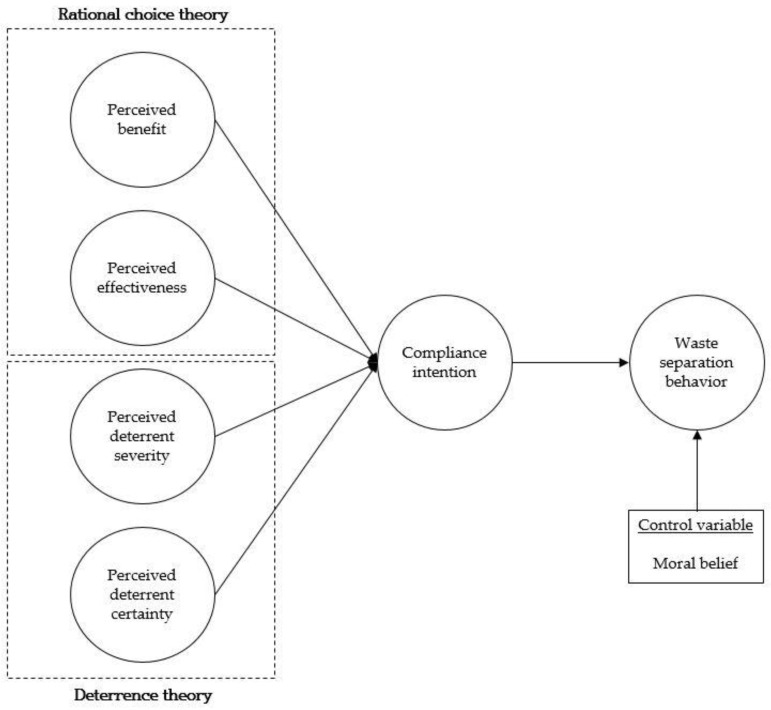
Research model.

Collectively, the literature review led us to propose the research model in Figure 1.

## 3. Methodology

### 3.1. Survey and Measurement Items

The research constructs were measured using pre-existing measurements from prior research and adapted to the research context with slight rewording. All scales were operationalized as reflective constructs and measured using five-point Likert scales. The specific items for measuring each construct and their sources are shown in Appendix A.

As a dependent variable, compliance behavior was measured using items from Goh et al. [2]. Compliance intention was the modification of those developed by Bulgurcu et al. [55] and Wang et al. [1]. Measures of the perceived benefit of MEP were drawn from Cudjoe et al. [45] and Nguyen et al. [10]. The measurement of perceived effectiveness of MEP was the modification of those developed by Cudjoe et al. [45], Liu et al. [44], and Wan et al. [47]. The items used for perceived deterrent severity and perceived deterrent certainty were developed by Son [56] and Xu et al. [49]. As a control variable, the items for moral belief were adapted from D’Arcy and Lowry [52]. Several demographic variables were included: gender, age, education level, and average monthly income.

Because both independent variables and dependent variable data were obtained from a survey, the variance inflation factor (VIF) values were examined to assess whether common method bias (CMB) was indeed a problem in our sample [57]. The test results showed that the VIF values ranged from 1.219 to 1.534, far below the recommended threshold of 3.3 [58], suggesting that CMB was not a problem for the data (see Appendix B).

### 3.2. Data Collection

The primary purpose of this study is to explore the antecedents of compliance intention in terms of waste separation policy and its consequences in the context of municipal solid waste management in South Korea. Because the total population is limited, this study aims to gain the views of only a targeted set of people based on location. The target population was households residing in apartment complexes in urban areas of South Korea because an apartment is a representative form of housing in South Korea due to higher population density. Doing a small pilot or exploratory research using non-probability sampling may be worthwhile to obtain more insights [59]. Thus, a non-probability-based sampling method for data collection was adopted.

The survey data to test the research model were collected from a panel of a professional market research firm. According to KOSIS (the Korean Statistical Information Service), the number of households in Seoul was 4,046,799 in 2021. The number of waste separation policy violations was 128,144 in 2021. The ratio of the non-compliance population was 3.2%. Thus, the household portion that complied with the waste separation policy was 96.8%. The minimum number of necessary samples to meet the desired statistical constraints is 48, meaning 48 or more measurements/surveys are needed to have a confidence level of 95% that the real value is within ±5% of the measured/surveyed value. A total of 306 responses were considered for analysis. Table 1 shows several demographic information about the respondents. There were 164 (53.59%) females and 142 (46.40%) males in the sample. In addition, 74.50% of the respondents received at least an undergraduate education. The author evaluated any systematic differences based on the demographic characteristics in the sample, and ANOVAs were performed for all research variables. For example, statistically significant differences in perceived benefit, perceived deterrent severity, and compliance intention were found among the education level group at the 0.05 level of significance (see Appendix C).

## 4. Results and Discussion

This study employed the PLS (partial least squares) technique to analyze the research model. Besides the advantages of the PLS technique, such as non-normal data and the use of formatively measured latent variables, the author chose PLS analysis because of the merit of small sample sizes [60]. Because the primary objective of this study was to predict rather than test an established theory, PLS analysis was considered suitable for this study [61].

### 4.1. Measurement Model Assessment

The author evaluated the reliability and validity of the measurements [62]. For construct reliability, as can be seen from Table 2, the Cronbach’s alpha values met the minimum threshold of 0.7, and all composite reliability (CR) was more significant than the recommended threshold of 0.7 [63,64]. 

For convergent validity, as shown in Appendix D, all individual items loaded highly on their intended construct. The factor loadings ranged from 0.700 to 0.954. In addition, as shown in Table 2, the average variance extracted (AVE) of all constructs exceeded the 0.5 thresholds [63,64]. 

For discriminant validity, the correlation between the indicator and its construct was higher than the correlation with other block constructs, demonstrating discriminant validity (see Appendix D). Moreover, the square root of the AVE should be larger than the inter-construct correlations [63]. As seen in the inter-construct correlations (off-diagonal elements) and the square root of the AVE (diagonal elements) in Table 3, the diagonal line of the correlation matrix exceeded the corresponding correlations with other factors. In addition, the author assessed the heterotrait–monotrait ratio of correlation (HTMT) approach [65]. As shown in Appendix E, the HTMT criterion, which refers to the average correlations of the indicators across constructs, fulfills the conservative threshold of 0.85. These results supported the convergent and discriminant validities of our measures.

### 4.2. Structural Model Assessment

**Figure 2:**
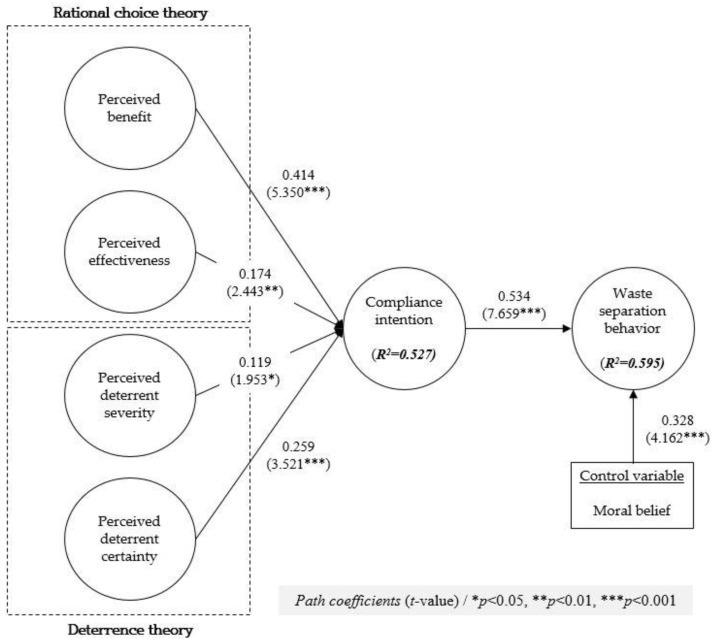
Results of path analysis.

This study employed the PLS bootstrapping technique with 5000 subsamples [66]. Figure 2 suggests the path coefficients and explains variances for the research model. The explained variances (*R*^2^) value in Figure 2 indicates that the structural model of this study explained 52.7% of the variance in compliance intention and 59.5% of the variance in waste separation behavior.

Regarding rational choice theory, the results of this study showed that perceived benefit significantly influenced compliance intention (β = 0.414, *p* < 0.001) and perceived effectiveness significantly affected compliance intention (β = 0.174, *p* < 0.01). Concerning deterrence theory, the results showed that perceived deterrent severity had a significant positive relationship with compliance intention (β = 0.119, *p* < 0.05) and perceived deterrent certainty had a significant positive influence on compliance intention (β = 0.259, *p* < 0.001). For antecedents of compliance behavior, compliance intention had a significant positive effect on waste separation behavior (i.e., compliance behavior) (β = 0.534, *p* < 0.001). In addition, as a control variable, moral belief positively affected waste separation behavior (β = 0.328, *p* < 0.001). Table 4 summarizes the results of the hypotheses testing.

### 4.3. Discussion

Regarding the benefits of waste separation behavior, the study shows that waste separation policy (WSP) compliance intention is motivated by the perceived benefit and perceived effectiveness of WSP. The effect on WSP is exerted mainly through perceived benefit rather than perceived effectiveness. The lack of statistical significance of perceived benefit concurs with the findings of previous studies in waste separation behavior [44]. The results suggest that perceived benefit motivates individuals to engage in waste separation behavior, even if the effectiveness of the waste separation behavior is perceived as being relatively low by individuals.

Regarding the cost of noncompliance with WSP, the results show that perceived deterrent severity and certainty influence WSP compliance intention. The deterrence effect is primarily exerted through perceived deterrent certainty rather than perceived deterrent sanction severity. The lack of statistical significance of perceived deterrent severity concurs with the findings of previous studies on compliance behavior [49]. The results suggest that individuals do not perceive the corresponding noncompliance sanctions with WSP to be severe. 

Consistent with the WSP literature, the results provide empirical evidence that WSP compliance intention positively affects waste separation behavior. In addition, as a control variable, moral belief significantly affects waste separation behavior. This result was in line with the findings from previous studies, which found that greater moral belief makes individuals more likely to engage in waste separation behavior.

## 5. Conclusions

### 5.1. Theoretical Implications

This study has several research implications for individual compliance with WSP. First, the literature on WSP is mainly based on norm-activation theory (NAT), value–belief–norms (VBN) theory, and the theory of planned behavior (TPB). Few studies have been devoted to an integrated view of individuals’ compliance with WSP based on rational choice theory [12,13] and deterrence theory [14,15]. Based on these theories, this study employed an integrated view of individuals’ compliance with WSP. Second, this study found that WSP compliance intention is influenced by perceived benefit and perceived effectiveness of waste separation behavior. This study found that the perceived benefits of waste separation behavior dominate the severity and certainty of perceived deterrence in individual decision calculus. As a result, the deterrence antecedents are less effective than the self-control antecedents in conducting waste separation behavior. Third, the results of this study also suggest that the perceived severity and certainty of a deterrence influence WSP compliance intention directly. Although some studies on mandatory policies showed that a deterrence’s perceived severity and certainty have no direct effect on compliance intention [8], this study found that the perceived deterrent severity and perceived deterrent certainty promote WSP compliance intention. These results are consistent with earlier studies on waste separation behavior [4,38].

### 5.2. Practical Implications

This study has significant implications for policymakers. First, this study shows that the perceived benefit and effectiveness of waste separation behavior motivates individuals to separate waste in their daily lives. Thus, it is necessary to provide more information related to waste separation behavior, such as the benefits and value of waste separation [1]. Providing pro-environmental performance indicators could encourage individuals’ WSP compliance intention [44]. In 2013, South Korea implemented universal curbside composting, forcing everyone to separate their food from general waste. Seoul has introduced automated food waste collectors in apartment complexes, which allow residents to forgo the food waste and swipe a card to pay the weight-based fee at the machine directly. The local government collects the revenue from the weight-based fee to help defray the costs of this process, effectively working as a pay-as-you-throw tax. Second, governments around the globe force individuals to separate household waste. A mandatory waste management policy can be a deterrent [4]. Providing a signal to the public about the severity and certainty of deterrence would prompt them to comply with WSP [5]. Third, individuals with firm moral beliefs could engage in waste separation behavior. Moral beliefs can be strengthened by developing public marketing campaigns and cultivating new pro-environmental paradigms in ordinary people through education. For example, South Korea funds environmental education and public relations campaigns and provides tools to local community members for managing their waste. Children’s education on waste, recycling, and reuse starts in kindergarten.

### 5.3. Limitations and Future Research

The study had some limitations that should be addressed in future research. First, this study used cross-sectional data to examine the hypotheses. A prospective study should consider longitudinal studies or other research methods, such as the field studies method, to validate the findings of this study. Second, this study includes a limited unit of analysis. A future study should consider adding waste separation behavior in workplaces to contribute more evidence to the existing waste separation behavior studies. Third, the study sample consists of citizens of Seoul, Korea. A future study with a broader sample size should be conducted in other cities and countries to generalize the findings. Fourth, waste separation behavior is affected by contextual factors such as media use [67]. Information from the media can be a vital contextual force in influencing the perceived benefits of waste separation behavior. Future studies should incorporate various contextual factors to enhance the results [17].

## Figures and Tables

**Table 1:** Demographic characteristics of sample.

Items	Category	Frequency	Ratio (%)
Gender	Female	164	53.59
	Male	142	46.40
Age	Under 30	32	10.45
	30–39	94	30.71
	40–49	104	33.98
	50–59	44	14.37
	60 or above	32	10.45
Education level	High school or below	50	16.33
	Bachelor’s degree	228	74.50
	Graduate school or above	28	9.15
Average monthly income (KRW)	Below 2,000,000	28	9.15
	2,000,000–2,999,999	40	13.07
	3,000,000–3,999,999	62	20.26
	4,000,000–4,999,999	90	29.41
	5,000,000–5,999,999	66	21.56
	Above 6,000,000	20	6.54

**Table 2:** Reliability of constructs.

Constructs	Mean (SD)	Cronbach’s Alpha	Composite Reliability	Average Variance Extracted (AVE)
Perceived benefit	3.289 (1.026)	0.858	0.905	0.705
Perceived effectiveness	2.842 (0.834)	0.829	0.887	0.663
Perceived deterrent severity	2.839 (0.883)	0.867	0.918	0.788
Perceived deterrent certainty	3.440 (0.882)	0.834	0.900	0.750
Compliance intention	3.632 (0.910)	0.911	0.944	0.849
Waste separation behavior	3.490 (0.837)	0.922	0.950	0.865
Moral belief	3.180 (0.871)	0.852	0.931	0.871

**Table 3:** Correlation matrix and AVEs.

Constructs	PB	PE	DS	DC	CI	CB	MB
Perceived benefit	**0.839**						
Perceived effectiveness	0.491	**0.815**					
Perceived deterrent severity	0.316	0.387	**0.888**				
Perceived deterrent certainty	0.432	0.318	0.082	**0.866**			
Compliance intention	0.649	0.505	0.338	0.503	**0.922**		
Waste separation behavior	0.506	0.552	0.408	0.368	0.723	**0.930**	
Moral belief	0.464	0.708	0.321	0.342	0.576	0.636	**0.933**

Legends: PB = perceived benefit, PE = perceived effectiveness, DS = perceived deterrent severity, DC = perceived deterrent certainty, CI = compliance intention, CB = compliance behavior, and MB = moral belief. Figures along the diagonal in bold are values of the squared root of the AVE.

**Table 4:** Summary of hypotheses testing.

Hypothesis	Path Coefficient	t-Value	*p*-Value	Results
Perceived benefit -> Compliance intention	0.414	5.350	0.000	Supported
Perceived effectiveness -> Compliance intention	0.174	2.443	0.007	Supported
Perceived deterrent severity -> Compliance intention	0.119	1.953	0.025	Supported
Perceived deterrent certainty -> Compliance intention	0.259	3.521	0.000	Supported
Compliance intention -> Waste separation behavior	0.534	7.659	0.000	Supported
Moral belief -> Waste separation behavior	0.328	4.162	0.000	Supported

## Data Availability

The data that support the findings of this study are available from the authors upon reasonable request.

## Appendix A

**Table A1:** Measurement items.

Constructs	Items	Items	Sources
Perceived benefit	PB1	I believe that waste separation helps reduce disposal of waste in landfills.	[10,45]
	PB2	I believe that waste separation helps reduce negative impacts on the environment.	
	PB3	My waste separation behavior will have an important educational effect on my children.	
	PB4	Waste separation has positive effects on residents’ perceptions in saving resources and protecting the environment.	
Perceived effectiveness	PE1	I am aware of how my waste separation behavior impacts the environment.	[44,45,47]
	PE2	I am willing to engage in waste separation behavior, even if it is inconvenient for me.	
	PE3	I am willing to make personal sacrifices to enhance the quality of the environment, even if the results do not seem so meaningful at the moment.	
	PE4	I believe that some of my waste separation behavior would enhance the quality of the living environment in our surroundings.	
Perceived deterrent severity	DS1	If I were caught violating the municipal waste separation policy, the sanctions would be very severe.	[49,56]
	DS2	My community would take strict action against violation of the municipal waste separation policy.	
	DS3	If I violate the municipal waste separation policy, the sanctions would put me in serious trouble.	
Perceived deterrent certainty	DC1	My community strictly enforces the municipal waste separation policy with residents.	[49,56]
	DC2	I am likely to incur sanctions if I violate the municipal waste separation policy.	
	DC3	My community explicitly communicates that sanctions will follow if the municipal waste separation policy is violated.	
Compliance intention	CI1	I intend to comply with the requirements of the municipal waste separation policy in my daily lives.	[1,55]
	CI2	I intend to protect environment according to the municipal waste separation policy in my daily lives.	
	CI3	I intend to carry out my responsibilities prescribed in the municipal waste separation policy in my daily lives.	
Waste separation behavior (Compliance behavior)	CB1	I put only general waste into the volume-based garbage bag.	[2]
	CB2	I separate paper, metal, glass, plastics and other recyclable waste from general waste and green waste.	
	CB3	I separate green waste from recyclable waste and general waste.	
Moral belief	MB1	I would find it morally unacceptable to violate the municipal waste separation policy.	[52]
	MB2	It would be against my moral beliefs to violate the municipal waste separation policy.	

## Appendix B

**Table A2:** Collinearity statistics (VIF).

Construct	PB	PE	DS	DC	CI	CB	MB
Perceived benefit					1.534		
Perceived effectiveness					1.467		
Perceived deterrent severity					1.219		
Perceived deterrent certainty					1.266		
Compliance intention						1.497	
Waste separation behavior							
Moral belief						1.497	

Legends: PB = perceived benefit, PE = perceived effectiveness, DS = perceived deterrent severity, DC = perceived deterrent certainty, CI = compliance intention, CB = compliance behavior, and MB = moral belief.

## Appendix C

**Table A3:** Results of ANOVA test.

		Sum of Squares	df	Mean Square	F	Sig.
Perceived benefit	Between groups	8.779	2	4.389	4.273	0.015
	Within groups	311.250	303	1.027		
	Total	320.029	305			
Perceived effectiveness	Between groups	4.072	2	2.036	2.975	0.053
	Within groups	207.366	303	0.684		
	Total	211.438	305			
Perceived deterrent severity	Between groups	15.997	2	7.999	10.969	0.000
	Within groups	220.938	303	0.729		
	Total	236.935	305			
Perceived deterrent certainty	Between groups	3.683	2	1.842	2.394	0.093
	Within groups	233.052	303	0.769		
	Total	236.735	305			
Compliance intention	Between groups	5.662	2	2.831	3.487	0.032
	Within groups	246.011	303	0.812		
	Total	251.673	305			
Waste separation behavior	Between groups	2.704	2	1.352	1.949	0.144
	Within groups	210.211	303	0.694		
	Total	212.915	305			
Moral belief	Between groups	2.503	2	1.251	1.662	0.191
	Within groups	228.111	303	0.753		
	Total	230.614	305			

## Appendix D

**Table A4:** Item factor loadings and cross-loadings.

Construct	Item	PB	PE	DS	DC	CI	CB	MB
Perceived benefit	PB1	**0.896**	0.450	0.232	0.326	0.591	0.443	0.426
	PB2	**0.896**	0.411	0.249	0.316	0.562	0.428	0.379
	PB3	**0.804**	0.452	0.248	0.473	0.560	0.435	0.397
	PB4	**0.752**	0.322	0.353	0.337	0.452	0.392	0.349
Perceived effectiveness	PE1	0.390	**0.854**	0.307	0.192	0.355	0.399	0.576
	PE2	0.390	**0.854**	0.409	0.187	0.395	0.484	0.541
	PE3	0.454	**0.840**	0.287	0.378	0.492	0.451	0.624
	PE4	0.349	**0.700**	0.259	0.241	0.375	0.459	0.551
Perceived deterrent severity	DS1	0.257	0.333	**0.929**	0.116	0.317	0.394	0.293
	DS2	0.223	0.288	**0.841**	0.074	0.233	0.280	0.258
	DS3	0.346	0.395	**0.891**	0.031	0.334	0.395	0.300
Perceived deterrent certainty	DC1	0.353	0.307	0.048	**0.845**	0.382	0.303	0.322
	DC2	0.410	0.291	0.089	**0.900**	0.471	0.338	0.320
	DC3	0.356	0.233	0.072	**0.852**	0.446	0.313	0.251
Compliance intention	CI1	0.573	0.420	0.300	0.455	**0.911**	0.676	0.516
	CI2	0.615	0.483	0.329	0.495	**0.954**	0.687	0.566
	CI3	0.605	0.494	0.304	0.440	**0.898**	0.636	0.510
Waste separation behavior	CB1	0.471	0.544	0.394	0.442	0.634	**0.936**	0.620
	CB2	0.473	0.486	0.352	0.332	0.653	**0.939**	0.592
	CB3	0.469	0.508	0.393	0.237	0.622	**0.915**	0.559
Moral belief	MB1	0.425	0.656	0.322	0.277	0.524	0.573	**0.929**
	MB2	0.439	0.666	0.279	0.359	0.551	0.612	**0.938**

Legends: PB = perceived benefit, PE = perceived effectiveness, DS = perceived deterrent severity, DC = perceived deterrent certainty, CI = compliance intention, CB = compliance behavior, and MB = moral belief.

## Appendix E

**Table A5:** Heterotrait–monotrait ratio (HTMT).

Construct	PB	PE	DS	DC	CI	CB	MB
Perceived benefit							
Perceived effectiveness	0.573						
Perceived deterrent severity	0.367	0.451					
Perceived deterrent certainty	0.510	0.371	0.097				
Compliance intention	0.731	0.573	0.373	0.573			
Waste separation behavior	0.570	0.630	0.448	0.413	0.786		
Moral belief	0.541	0.839	0.372	0.406	0.653	0.715	

Legends: PB = perceived benefit, PE = perceived effectiveness, DS = perceived deterrent severity, DC = perceived deterrent certainty, CI = compliance intention, CB = compliance behavior, and MB = moral belief.
